# History matters: ecometrics and integrative climate change biology

**DOI:** 10.1098/rspb.2010.2233

**Published:** 2011-01-12

**Authors:** P. David Polly, Jussi T. Eronen, Marianne Fred, Gregory P. Dietl, Volker Mosbrugger, Christoph Scheidegger, David C. Frank, John Damuth, Nils C. Stenseth, Mikael Fortelius

**Affiliations:** 1 Department of Geological Sciences, Indiana University, 1001 East 10th Street, Bloomington, IN 47405, USA; 2 Department of Geosciences and Geography, University of Helsinki, PO Box 64 (Gustaf Hällströmin katu 2a), 00014 Helsinki, Finland; 3 Coastal Zone Research Team, ARONIA Research Institute at Åbo Akademi University and Novia, University of Applied Sciences, Raseborgsvägen 9, 10600 Ekenäs, Finland; 4 Paleontological Research Institution, 1259 Trumansburg Road, Ithaca, NY 14850, USA; 5 Senckenberg Forschungsinstitut und Naturmuseum, Senckenberganlage 25, 60325 Frankfurt, Deutschland; 6 Swiss Federal Research Institute WSL, Zürcherstrasse 111, 8903 Birmensdorf, Switzerland; 7 Oeschger Centre for Climate Change Research, University of Bern, Zähringerstrasse 25, 3012 Bern, Switzerland; 8 Department of Ecology, Evolution and Marine Biology, University of California, Santa Barbara, CA 93106, USA; 9 Centre for Ecological and Evolutionary Synthesis (CEES), Department of Biology, University of Oslo, PO Box 1066, Blindern 0316, Oslo, Norway; 10 Institute of Biotechnology, University of Helsinki, PO Box 56 (Viikinkaari 9), 00014 Helsinki, Finland

**Keywords:** climate change, scalability, traits, ecometrics, species interactions

## Abstract

Climate change research is increasingly focusing on the dynamics among species, ecosystems and climates. Better data about the historical behaviours of these dynamics are urgently needed. Such data are already available from ecology, archaeology, palaeontology and geology, but their integration into climate change research is hampered by differences in their temporal and geographical scales. One productive way to unite data across scales is the study of functional morphological traits, which can form a common denominator for studying interactions between species and climate across taxa, across ecosystems, across space and through time—an approach we call ‘ecometrics’. The sampling methods that have become established in palaeontology to standardize over different scales can be synthesized with tools from community ecology and climate change biology to improve our understanding of the dynamics among species, ecosystems, climates and earth systems over time. Developing these approaches into an integrative climate change biology will help enrich our understanding of the changes our modern world is undergoing.

## 1. Introduction

Anthropogenic climate change is an established reality: many of the remaining questions are about its magnitudes and impacts [1–3]. The interactions between changing climate and biotas are of especial interest, and it is important to understand whether current changes are unprecedented or comparable to past events from which we can better understand what lies ahead. The geographical ranges of plants, birds and butterflies, for example, have been pushing northward by more than 10 km per decade as the global climate has warmed in the late 20th century [4–6]. Are these changes similar to the transition from the Medieval Climatic Optimum to the Little Ice Age [7], the transition from the Late Glacial to the Early Holocene [8,9] or the major oscillations in Earth's climate that occurred deeper in the geological past, many of which caused massive biotic reorganization and extinction [10,11]? The changes Earth is about to experience will almost certainly be greater than any experienced in human history, probably greater and certainly different than any change in the last 2 Myr, which means we need to look to deeper time for informative comparisons. We are in urgent need of a historical context in which to place such observations in order to better inform near-future predictions.

Geohistorical records provide that context. Data from long-range ecological studies, archaeology, palaeontology and geology record how species have responded to changing climates, how ecological communities have assembled and reassembled, how some dynamics have led to mass extinctions and some have not, and how feedbacks between climate and biota have driven and ameliorated climate change. Importantly, historical data allow rates of change to be measured over the broad temporal and geographical scales at which climate operates [8,12–14]. But despite their common interest in the dynamics between life and climate, conservation biologists, ecologists, niche modellers, climate modellers, palaeontologists and geologists tend to measure different variables at scales that may differ by orders of magnitude [15].

## 2. Ecometrics: the analysis of functional traits

**Figure 1.**
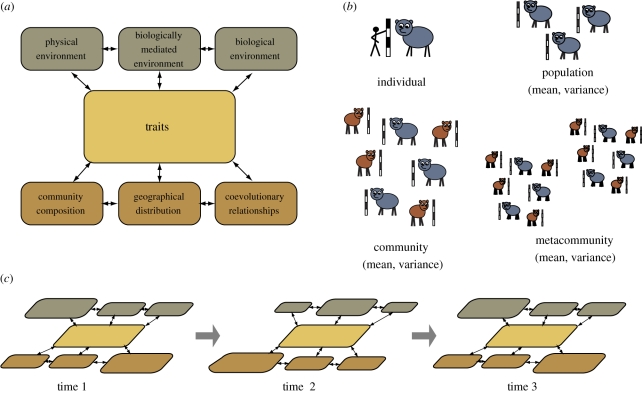
(*a*) Environmental, ecological and geographical aspects of biotic change are connected through traits. One way of measuring change in a biotic system is thus by measuring ecometric traits. (*b*) Ecometric traits can be properties of an individual, of a population and of a community, or even of some larger level of organization. The interactions shown in (*a*) can involve traits on any or all of these levels. (*c*) The system of interactions itself evolves as changes in one part of the system feed back to the others.

Organismal traits are one promising way to integrate data across time and space—specifically, traits that are functionally related to the organism's physical (e.g. climate), biological (e.g. macrovegetation) or biologically mediated environment (e.g. the sheltered microclimates below the canopy of a dense forest; figure 1*a*). Traits such as leaf shape and tooth structure mediate interactions between organisms and their surroundings [16,17], and thus determine the place and circumstances in which the organism can most productively live. Furthermore, the environments to which the organism is exposed result in selection on those traits. Traits are thus central to the differential survival and reproduction of individuals in different environmental and geographical contexts. The cumulative effects of traits in the individuals of a population influence where its members flourish, which influences the total geographical distribution of species, at both small and large scales. The cumulative effect of traits across species therefore feeds up into the assembly and dissolution of communities [18–21]. Traits are thus a central mechanism in geographical range shifts and community restructuring, and are therefore useful for studying the feedbacks between biota and climate. For example, Köppen's classic climate classification was trait-based in its use of vegetation phenology as a proxy for the combination of precipitation and temperature [22].

For the traits to be a useful bridge between modern, ecological, archaeological and palaeontological contexts, they must be measurable from fossil remains and be relevant to important climatic and environmental factors. In such cases, it can be used as a proxy for the dynamic interaction between organisms and environments, an approach we refer to as ‘ecometrics’ [23]. Several ecometric traits are already being studied, many of which relate to environmental variables of broad interest to climate change biology (table 1). Functional trait data and the methods available for analysing them are growing rapidly [24–27]. The promise of functional traits has already been seen by ecologists [24,25,28–30] and palaeontologists [16,31–35]: we see traits as an opportunity to bridge these disciplines for the study of climate change biology.

**Table 1.** Examples of ecometric traits that can be applied to modern and fossil organisms.

**Figure 2.**
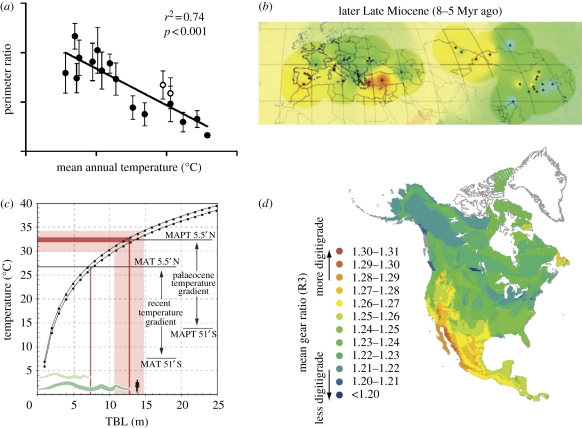
Examples of ecometrics. (*a*) The ratio of leaf perimeter to leaf area in deciduous plants is correlated with mean annual temperature and can be used to estimate temperature from leaf community assemblages (adapted from [16]). (*b*) Average tooth hypsodonty in mammalian herbivores is correlated with precipitation and coarseness of vegetation. This map of mean hypsodonty in Miocene faunas has been used to reconstruct precipitation patterns in Eurasia (adapted from [32]). (*c*) Ambient temperature influences the range of size of poikilothermic animals in a community [99], allowing the size range of fossil snakes to be used as a ‘palaeothermometer’ (adapted from [33]). (*d*) Average locomotor proportions of the calcaneum from the ankle of mammalian carnivores are correlated with ecoregion, as this map of mean proportions in North American carnivoran communities shows (after [71]).

*Leaf physiognomy*. The average shape of leaf serrations and lobes in dicot communities are related to mean annual temperature and water stress [59,97] (figure 2*a*). These traits are used to estimate continental climate conditions during the Mesozoic and Cenozoic from fossil floras [16,98–100].
*Leaf venation density*. The density of veins in the leaves of seed plants is related to transpiration and water availability, and it has been used to estimate these parameters from the Carboniferous to the present day [101].
*Stomatal density*. The density of stomatal pores on the surfaces of plant leaves and stems, through which carbon dioxide and oxygen are exchanged with the atmosphere, is inversely related to atmospheric CO_2_ concentration [38]. Stomatal density measured from fossil leaves tracks CO_2_ concentrations through the industrial era [102] and geological history [103].
*Ectothermic body size*. Metabolic rate decreases as body mass increases. Organisms cannot function with mass-specific metabolic rates below a certain threshold, placing a limit on the maximum size they can attain. In poikilotherms, whose internal temperature varies with the surrounding environment, mass-specific metabolic rate increases with ambient temperature, meaning that the maximum attainable size varies with environmental temperature [104,105]. The maximum size of terrestrial poikilotherms is a trait that has been used to estimate palaeotemperature [33] (figure 2*c*).
*Limb proportions*. The proportion of limb segments is related to stride length, speed and power in terrestrial vertebrates [106]. Arboreality, cursoriality and other locomotor styles differ in limb proportions. Because different macroenvironments favour different locomotor styles, average limb proportions in mammalian communities vary with macrovegetation and ecological region [71] (figure 2*d*).
*Body mass*. Body mass is related to ambient temperature, metabolic rate, substrate, diet and many life-history variables [107]. The analysis of body size in relation to mean annual temperature and macrovegetation is a well-developed ecometric example [34,108,109].
*Tooth crown complexity*. The shapes of the occluding surface of mammalian cheek teeth are specialized for food processing. The number of surface patches with the same occlusal orientation is smaller in carnivorous than in omnivorous and herbivorous teeth, making the ‘patchiness’ of the tooth crown highly correlated with the proportion of vegetation in the diet [17].

The key to the ecometric approach is identifying specific trait–environment pairings and using those traits to study the dynamics of the pairing across space and time. Vegetative traits such as leaf shape and stomatal counts are closely tied to the ratio of evapotranspiration to precipitation, which is an important driver of soil moisture and therefore an important factor in ecosystem organization [36,37]. This ratio can be hard to estimate from climate modelling because it is sensitive to factors such as soil type, shade and ground cover, but it is relatively easy to estimate from ecometric trait analysis. Such ecometric data therefore help establish the long-term history of some of the boundary conditions needed for climate modelling and other kinds of climate change science, and they allow the organism–trait–climate relationship to be studied in its own right.

## 3. Matters of scale

Ecometric traits are scalable in that they can be measured in individuals, populations, species, guilds, communities or metacommunities [23] (figure 1*b*). For example, the same trait can be used to measure the plastic changes in an individual, to characterize the common features of a plant biome and to measure the rate of escalation in predator–prey defences over geological time. By understanding the trait patterns at each of these scales, processes operating at each scale can be linked via the trait. Thus, traits provide a common denominator for linking data across hierarchies of scale and studying the interplay of processes operating at different levels in the hierarchy.

The dynamics between changing climates and biotas is most obvious at large scales, and ecometrics is arguably at its best at those same scales. The trait–environment relationship is most obvious at the community scale because the phenotypic variety and range of environments associated with a single population are normally small and difficult to measure. Individual genetic and life-history variation may mask the relationship in populations, but when ecometric data are averaged across species in a community and examined among communities across broad geographical scales (or across deep palaeontological time), the relationship becomes clearer because the quirks of individuals and populations are smoothed out [38,39].

## 4. Traits and people

Ecometric traits can also be a key to understanding how climate change will affect societies and cultures [40]. Traits are what we value or disdain in organisms—the structural traits of woods, the chemical traits of herbs, the locomotor traits of work animals, the disease-carrying traits of pests or the terrifying traits of large carnivores—and they influence the cultural priorities we place on cultivating, conserving or extinguishing species [41]. As we modify the traits and geographical distributions of species whose traits resonate with ours, we are in turn modifying the mosaic of (co-)evolutionary interactions, community compositions and geographical distributions, generating feedback loops that are a dominant part of the dynamics of the world's climate and biotic systems [42].

## 5. Ecometric tools

The tools needed to integrate climatic and biotic data over different temporal and spatial scales are still underdeveloped. We know, for example, the rate at which the geographical ranges of species are changing today over years or decades [5,43] and we can estimate the magnitude of geographical changes in the fossil record that happened over tens or hundreds of thousands of years [44]. One key to understanding current climate change is to know whether the rates today extrapolate into the shifts observed in the past and, therefore, whether the associated changes to past ecosystems are a likely result of today's climate change. Ecometrics can be developed into a tool to help integrate data across these scales.

Functional traits themselves do not solve the problems of scale, but the techniques used to study trait change in the fossil record combined with those commonly used in climate change biology may help. The precision of data collected from ecological studies and fossil samples are quite different, sometimes by orders of magnitude. Statistical techniques such as rarefaction, randomization and bootstrapping provide one avenue for making cross-scale comparisons [45–47]. Modelling processes at fine-scale resolution and testing the predictions of those models against data taken from larger temporal and geographical scales are also key to integrating across disciplines and scales [48,49]. Using palaeontological techniques, such as subsampling and binning, to standardize data will necessarily coarsen the spatial and temporal precision of data collected at ecological scales, but this coarsening can be advantageous because many of the patterns and processes associated with organism–climate interactions only manifest themselves on larger scales that are unaffected by the amalgamation of data [15,39]. Sampling to a palaeontological scale gives us an accurate picture of the long-term average behaviour of biotic systems, which usefully reduces the complexity of the data.

However, we must pay special attention to determining whether ecometric trait distributions reflect the same biological processes when observed at different spatial or temporal scales. Tree ring analyses provide relevant examples of how such patterns depend upon scale and can also provide illustrations of some of the complexities that may be observed as the ecometric approach develops. Their wide geographical distribution, their annual resolution and the normally high correlation between ring width, latewood density, isotopic composition and climate have made tree rings one of the most important proxies in assessing regional to hemispheric change over the past centuries to millennia [50]. But, coupled with these advances, this field has also uncovered some of the difficulties in applying biological metrics to make inferences of climate variation. For example, a narrow ring generally signifies cold temperatures in trees growing at the elevational or latitudinal tree lines, yet a similarly narrow ring can be indicative of drought stress in trees growing away from its low thermal growth limit [51]. Between these two extremes, simple interpretation may start to break down, providing a theoretical framework for the so-called ‘divergence problem’—a possible reduction in the degree to which tree-ring-based temperature reconstruction actually reflects temperature—within a warming planet [52–54]. Furthermore, interactions within ecological systems, such as between insects and their tree hosts, can leave non-climatic fingerprints on growth [55]. The complexity of the systems and the multitude of processes may result in complicated ‘emergent’ properties that may be difficult to disentangle even with a fairly complete knowledge of the system [56]. Such emergent properties may include uniquely characteristic responses of individual species, such as hemlock trees showing positive correlations with March temperatures in the year prior to ring formation [56,57]. Better developing the ecometric approach will help us better understand which patterns are associated with long-term, large-scale processes and which are local at both the species and community levels.

## 6. Taxon-specific analysis and ecometrics

Where the goal is to understand a single species, a taxon-specific ecometric analysis can greatly improve our understanding of how species will respond to a wide range of climatic conditions unlike those of the present day. For example, the Late Holocene fossil record of the tiger salamander (*Ambystoma tigrinum*) from Lamar Cave in Yellowstone National Park, Wyoming, was used to assess responses in morphology and life history to changes in climate [58]. Experimental studies with living tiger salamanders indicate that this species is able to exploit alternative life histories in response to environmental conditions. Tiger salamanders can either metamorphose into a terrestrial adult or remain aquatic and retain a paedomorphic (larval) morphology. *Ambystoma tigrinum* increased in body size in response to the largest climatic shift in the Yellowstone region over the last 3000 years, the Medieval Climatic Optimum (AD 800–1300, or 1150–650 years BP). There was also no trend in the ratio of paedomorphic to metamorphic individuals, indicating that not all life-history traits responded to climate changes. Such an approach is a valuable tool for the study of climate–organism interactions, but it is usually limited to the last 1 or 2 Myr of Earth history because of its ‘taxon-specific’ nature: in other words, the link between climate and organisms is based on the taxonomic identity of species in the modern world whose relationship to climate or environment is known.

## 7. Taxon-free analysis and ecometrics

A promising aspect of the ecometric approach is its potential for taxon-free analysis, thus allowing systems to be compared that do not share the same taxa, a critical requirement for comparing changes in the modern world to those in deep time. Because the focus is on traits, ecometric studies can proceed entirely by analysis of trait distributions independent of taxonomic nomenclature. Focusing on traits whose functions are directly related to environment adds to the generality of results, allowing them to be applied to any system in which organisms possess those traits regardless of the scale of analysis [26,28,32,59]. For taxon-free analysis to be successful, it should be based on trait systems in which the function–environment relationship is general enough to apply to any taxon in which the trait is found. The trait–environment relationship can then be quantified using transfer functions (equations that predict an environment based on the mean state of a trait; e.g. [60,61]), using performance filters (which measure how well traits perform in different environments [23]) and using performance currencies (which measure the biological performance of organisms in different environments, usually in terms of their ability to acquire resources [26]). It should be noted that even trait-based methods are not completely taxon-free because traits arise phylogenetically and are shared by particular clades, sometimes homoplastically. Occlusal complexity in cheek teeth, for example, is a trait that is specific to vertebrate animals, mostly mammals and dinosaurs, and cannot be applied to other taxa. Nevertheless, ecometric approaches can be applied broadly through time and space in a way that taxon-specific approaches cannot. The taxon-free approach is generalizable because it is based on the physical mechanics of trait–environment interactions, individual performance and population regulation, rather than the individual peculiarities of particular species.

## 8. Mammalian hypsodonty: a taxon-free ecometric example

**Figure 3.**
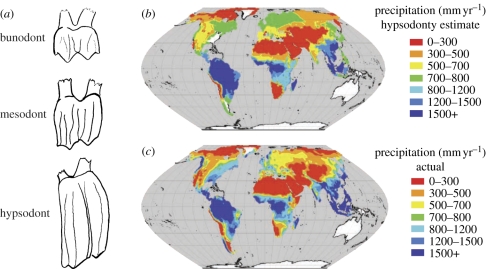
Hypsodonty and precipitation. (*a*) Cheek teeth of three ungulate species in lateral view. (*b*) Global precipitation estimated from the hypsodonty index of mammalian herbivore communities. (*c*) Actual global precipitation. (*b*,*c*) Adapted from [68].

An example of ecometric analysis that we have worked involves precipitation and hypsodonty, the high-crowned cheek tooth morphology possessed by many herbivores (figure 3*a*). Hypsodont teeth have evolved in many groups, including equids, bovids, murids, castorids, elephantids, macropodids, vombatids and others [62]. The structure, physiology and development of tooth crown height have been studied extensively [63–66], as have the relationships of hypsodonty to tooth function and diet [62]. The hypsodont crown is an adaptation to abrasive foodstuffs, such as airborne grit or silicaceous phytoliths, prolonging the functional life of the tooth against increased wear. Different diets vary in the amount of wear they produce: species that eat abrasive foods usually have high-crowned teeth that last a longer time to compensate for the high rate of wear [67]. The index of hypsodonty, or crown height, has been measured in fossil faunas to reconstruct changing patterns of aridity, which is associated with dietary abrasiveness [33,68,69] (figure 2*b*).

When the degree of hypsodonty is averaged across the species in mammalian herbivore guilds, there is a strong geographical correlation with precipitation, with higher-crowned species populating communities in arid, grassy regions where silica and other abrasives are commonly found in the local plants [33,70]. Eronen *et al*. [68] used regression trees to quantify the relationship and found that 65.8 per cent of the geographical variance in mean tooth crown height was explained by precipitation. They used the same regression tree to predict precipitation based on hypsodonty (figure 3*b*) and found good agreement with actual patterns of precipitation (figure 3*c*). Hypsodonty can thus be used to study the temporal and geographical dynamics among plant communities, herbivore communities and climate (figure 2*b*) [33,69]. These authors found that the shift to more arid conditions during the Late Miocene was associated with major restructuring of plant and herbivore communities across the globe, a transition that was not simultaneous everywhere, but which they tracked through space and time by mapping the hypsodonty index. The change in the ecometric trait of hypsodonty could thus be used to measure patterns of community reorganization, the magnitude and geographical pattern of the climatic change and the rates at which they occurred. These data are directly relevant to forecasting the possible severity of community reorganizations and geographical impact of current climatic change, given the existing scenarios for the rate of abiotic change over the next century.

## 9. Future directions

### (a) Trait–environment modelling

One of the prerequisites for ecometrics is to establish the relationship between trait and environment. Usually, this is done by regressing trait values on environmental variables [59,68,71]. We anticipate that the spatially explicit techniques used in habitat modelling (also known as species distribution modelling, niche modelling and bioclimate envelope modelling [72,73]) will be adapted to evaluate trait values at the level of both species and communities.

In habitat modelling, the geographical range of a species is used to extract climate data from any number of variables, such as mean annual temperature, annual precipitation and seasonality. The climate data associated with the species's range are used to construct a climate distribution or envelope, from which all the geographical areas with a climate compatible with that species can be identified. The same climate distribution has been projected onto past and future climate models to predict where that species will live (or did live) under different climate patterns [74–76]. Habitat modelling has the drawback that it measures the current association of a species with climate without knowledge of whether the species's distribution is limited by climate or by competition, geographical barriers or the chance of history, and therefore without knowledge of whether the species could tolerate a much wider range of climate [77–79]. Palaeontological and other historical data are emerging as an important line of evidence for testing whether the realized habitat of a species is coincident with its potential habitat [80,81], especially when the evolutionary changes one expects in the species-to-habitat relationship are taken into account [82,83].

Habitat modelling can be adapted to the study of traits in at least two ways: trait values can be substituted for species occurrences to map expected geographical shifts of ecometric patterns under different climate models, and the geographical range changes of entire communities of species can be modelled and the predicted change in ecometric patterns calculated for testing against real ecometric data. Embedding such analyses in multilevel models that include functional trait distributions, performance filters and filtered trait distributions will enhance the theoretical underpinnings and, perhaps, the predictive power of the models [23]. The technique was developed using modern species ranges and climate data, but the same methods have been adapted to geological data using fossil occurrences and climate proxy data such as isotopic measures of C_4_ vegetation, soil type, occurrences of climatically sensitive species, and isotopic measures of temperature and precipitation [84,85].

### (b) Community interactions

Species do not interact with climate in isolation: the interactions among species are fundamental to understanding the climate–organism dynamic, even in trait-based analyses. Changing climate can affect the dynamics between species, and the dynamics between species can affect the interaction of the species with climate. For example, if the boundary between the geographical ranges of two parapatric species is defined by competitive exclusion and both species experience climate change but respond differently, then one species may prevent the other from tracking its optimal habitat, resulting in unequal responses in the two species, one of which would not be the response predicted from the species–climate relationship alone [86]. The same conceptual approach is applicable to climate–organism dynamics in records on all scales, ecological and geological, and comparisons across scales will generate important insights into the short- and long-term consequences of dynamically interacting components in the Earth system.

The value of studying species interactions is almost self-evident, but how to apply ecometrics to that study is less obvious. These species–species interaction models could be adapted in a scalable taxon-free manner to interactions among ‘packages’ of traits in a community—traits that interact with one another yet respond differently to climate change. By ‘trait package’ we mean the combination of traits possessed by individual organisms or species: dental structures, limb structures or temperature regulation structures, for example. Some of these traits may interact with the traits of other species (e.g. traits associated with prey capture or foraging), some with the biotic environment (e.g. traits associated with moving through the vegetative substrate), some with abiotic climate (e.g. traits associated with insulation). Two interacting trait packages might interact with each other through one set of traits, but might have traits that interact differently with abiotic climate through the other. For example, today the Canadian lynx and snowshoe hare (a digitigrade carnivore and a hypsodont herbivore) undergo decade-long population cycles that are structured into three climatic regions because of the lynx's interaction with the hardness of snowpack [87]. Quantitative traits associated with foot load have been shown to be correlated with snow cover in both carnivores and herbivores [88,89]. These traits will interact similarly with abiotic climate in both groups. The dental traits of carnivores, however, are probably correlated with their prey, and do not carry environmental signal as such, whereas the dental traits of herbivores are probably correlated with vegetation cover, which is correlated to precipitation and temperature.

The simplification of a community into ‘modules’ [86] is a useful tool for scalability since it can be applied not only to the study of interactions of species or trait packages in the laboratory and the natural modern world, but also to historical data on ecological timescales (including data derived from museum voucher specimens collected over the last century or two) or to deep-time palaeontological data where complete communities can almost never be studied. A community module consists of a small number of species that strongly interact, such as predator–prey pairs or members of a trophic cascade. The interactions among species combine with the interactions between the individual species and climate in a dynamic that influences how the species, and therefore the community, respond to climate change. For example, the relationship between insect mouth parts, vegetative structures and climate has already been studied through much of the Phanerozoic [90–92]. On scales of hundreds of millions of years, the multiplication of functional classes of mouth parts coincided with major global changes in climate and plant diversity [91]. On shorter timescales associated with major events, like the Cretaceous–Tertiary extinction 65 Myr ago, insect functional diversity did not decrease appreciably, but specialized insect–plant associations dropped relative to more general ones [93].

More studies of the dynamics among communities, environments and climate are needed, especially ones that are scaled in terms of the rates of temporal change, the rates of spatial change and the magnitudes of the climate and trait changes. Such data will augment the critically needed baseline for forecasting the effects of current anthropogenic change [13,28].

## 10. Conclusion

Under the initiative of the International Union of Biological Sciences (IUBS), a group of ecologists, palaeontologists, palaeoanthropologists, modellers, climatologists and computer scientists were brought together in order to address these challenges focusing on the geobiological aspects of ‘integrative climate change biology’ (iCCB). The challenge we have set for ourselves is to develop the study of how biotic systems interact with changing climate, not only at present, but also, seamlessly, in the geological past. Complex interactions and feedback loops within the abiotic–biotic system and changing ecological networks need to be described so that patterns from many temporal and spatial scales can be integrated and their mechanisms understood. We intend our effort to be integrative to provide hierarchical explorations of processes at the individual, population and community levels [29,94,95]. We will need to understand the past if we are to forecast the future.

Climate change biology is a complex societal and scientific issue that requires joint efforts in scientific research, outreach and education. Only when researchers of diverse expertise join forces to (i) identify, articulate and structure the problem, (ii) provide hierarchical explorations of the issue, and (iii) develop research, outreach and educational frameworks, can we address climate change biology in a proper way [96]. Overcoming challenges of inter-disciplinary research requires a common framework and language that is able to link biological and physical processes that occur, and are investigated, across a huge variety of spatial and temporal scales.

## References

[1] IPCC 2007 Climate change 2007: synthesis report. Contribution of Working Groups I, II, and III to the Fourth Assessment Report of the Intergovernmental Panel on Climate Change (eds Pachauri R. K. Reisinger A.). Geneva, Switzerland: Intergovernmental Panel on Climate Change

[2] Rosenzweig C. 2008 Attributing physical and biological impacts to anthropogenic climate change. Nature 453, 353–357 10.1038/nature06937 (doi:10.1038/nature06937) 18480817

[3] Jones C. Lowe J. Liddicoat S. Betts R. 2009 Committed terrestrial ecosystem changes due to climate change. Nat. Geosci. 2, 484–487 10.1038/ngeo555 (doi:10.1038/ngeo555)

[4] Parmesan C. Yohe G. 2003 A globally coherent fingerprint of climate change impacts across natural systems. Nature 421, 37–42 10.1038/nature01286 (doi:10.1038/nature01286) 12511946

[5] Hickling R. Roy D. Hill J. Fox R. Thomas C. 2006 The distributions of a wide range of taxonomic groups are expanding polewards. Global Change Biol. 12, 450–455 10.1111/j.1365-2486.2006.01116.x (doi:10.1111/j.1365-2486.2006.01116.x)

[6] Loarie S. R. Duffy P. B. Hamilton H. Asner G. P. Field C. B. Ackerly D. D. 2009 The velocity of climate change. Nature 462, 1052–1055 10.1038/nature08649 (doi:10.1038/nature08649) 20033047

[7] Grove J. 1988 The Little Ice Age. London, UK: Methuen

[8] Barnosky A. Hadly E. Bell C. 2003 Mammalian response to global warming on varied temporal scales. J. Mammal. 84, 354–368 10.1644/1545-1542(2003)084<0354:MRTGWO>2.0.CO;2 (doi:10.1644/1545-1542(2003)084<0354:MRTGWO>2.0.CO;2)

[9] Koch P. Barnosky A. 2006 Late Quaternary extinctions: state of the debate. Ann. Rev. Ecol. Syst. 37, 215–230 10.1146/annurev.ecolsys.34.011802.132415 (doi:10.1146/annurev.ecolsys.34.011802.132415)

[10] McElwain J. Punyasena S. 2007 Mass extinction events and the plant fossil record. Trends Ecol. Evol. 22, 548–557 10.1016/j.tree.2007.09.003 (doi:10.1016/j.tree.2007.09.003) 17919771

[11] Erwin D. 2008 Extinction as the loss of evolutionary history. Proc Natl Acad. Sci USA 105, 11 520–11 527 10.1073/pnas.0801913105 (doi:10.1073/pnas.0801913105) 18695248 PMC2556409

[12] Erwin D. 2009 A call to the custodians of deep time. Nature 462, 282–283 10.1038/462282a (doi:10.1038/462282a) 19924193

[13] Willis K. J. Birks H. J. B. 2006 What is natural? The need for a long-term perspective in biodiversity conservation. Science 314, 1261–1265 10.1126/science.1122667 (doi:10.1126/science.1122667) 17124315

[14] Hadly E. A. Barnosky A. D. 2009 Vertebrate fossils and the future of conservation biology. In Conservation paleobiology: using the past to manage for the future (eds Dietl G. P. Flessa K. W.), pp. 39–60 New Haven, CT: The Paleontological Society

[15] McGill B. 2010 Matters of scale. Science 328, 575–576 10.1126/science.1188528 (doi:10.1126/science.1188528) 20431001

[16] Royer D. L. Wilf P. Janesko D. A. Kowalski E. A. Dilcher D. L. 2005 Correlations of climate and plant ecology to leaf size and shape: potential proxies for the fossil record. Am. J. Bot. 92, 1141–1151 10.3732/ajb.92.7.1141 (doi:10.3732/ajb.92.7.1141) 21646136

[17] Evans A. R. Wilson G. P. Fortelius M. Jernvall J. 2006 High-level similarity of dentitions in carnivorans and rodents. Nature 445, 78–81 10.1038/nature05433 (doi:10.1038/nature05433) 17167416

[18] Thompson J. 2005 The geographic mosaic of coevolution. Chicago, IL: University of Chicago Press

[19] Keddy P. 1992 Assembly and response rules: two goals for predictive community ecology. J. Veget. Sci. 3, 157–164 10.2307/3235676 (doi:10.2307/3235676)

[20] Poff N. 1997 Landscape filters and species traits: towards mechanistic understanding and prediction in stream ecology. J. N. Am. Benthol. Soc. 16, 391–409 10.2307/1468026 (doi:10.2307/1468026)

[21] Blois J. L. Hadly E. A. 2009 Mammalian response to Cenozoic climatic change. Annu. Rev. Earth Planet. Sci. 37, 181–208 10.1146/annurev.earth.031208.100055 (doi:10.1146/annurev.earth.031208.100055)

[22] Köppen W. 1931 Grundriss der klimakunde. Berlin, Germany: W. de Gruyter

[23] Eronen J. T. Polly P. D. Fred M. Damuth J. Frank D. C. Mossbrugger V. Scheidegger C. Stenseth N. C. Fortelius M. 2010 Ecometrics: the traits that bind the past and present together. Int. Zool. 5, 88–101 10.1111/j.1749-4877.2010.00192.x (doi:10.1111/j.1749-4877.2010.00192.x) 21392327

[24] Box E. O. 1996 Plant functional types and climate at the global scale. J. Veget. Sci. 7, 309–320 10.2307/3236274 (doi:10.2307/3236274)

[25] McGill B. Enquist B. Weiher E. Westoby M. 2006 Rebuilding community ecology from functional traits. Trends Ecol. Evol. 21, 178–185 10.1016/j.tree.2006.02.002 (doi:10.1016/j.tree.2006.02.002) 16701083

[26] Weiher E. Werf A. Thompson K. Roderick M. Garnier E. Eriksson O. 1999 Challenging Theophrastus: a common core list of plant traits for functional ecology. J. Veget. Sci. 10, 609–620 10.2307/3237076 (doi:10.2307/3237076)

[27] Webb C. T. Hoeting J. A. Ames G. M. Pyne M. I. Poff N. L. 2010 A structured and dynamic framework to advance traits-based theory and prediction in ecology. Ecol. Lett. 13, 267–283 10.1111/j.1461-0248.2010.01444.x (doi:10.1111/j.1461-0248.2010.01444.x) 20455917

[28] Thompson J. N. 2001 Frontiers of ecology. Bioscience 51, 15–24 10.1641/0006-3568(2001)051[0015:FOE]2.0.CO;2 (doi:10.1641/0006-3568(2001)051[0015:FOE]2.0.CO;2)

[29] Díaz S. Cabido M. 2001 Vive la difference: plant functional diversity matters to ecosystem processes. Trends Ecol. Evol. 16, 646–655 10.1016/S0169-5347(01)02283-2 (doi:10.1016/S0169-5347(01)02283-2)

[30] Westoby M. Wright I. 2006 Land-plant ecology on the basis of functional traits. Trends Ecol. Evol. 21, 261–268 10.1016/j.tree.2006.02.004 (doi:10.1016/j.tree.2006.02.004) 16697912

[31] Damuth J. D. Jablonski D. Harris R. M. Potts R. Stucky R. K. Sues H. D. Weishampel D. B. 1992 Taxon-free characterization of animal communities. In Terrestrial ecosystems through time: evolutionary paleoecology of terrestrial plants and animals (eds Beherensmeyer A. K. Damuth J. D. diMichele W. A. Potts R. Sues H. D. Wing S. L.), pp. 183–203 Chicago, IL: University of Chicago Press

[32] Fortelius M. 2002 Fossil mammals resolve regional patterns of Eurasian climate change over 20 million years. Evol. Ecol. Res. 4, 1005–1016

[33] Head J. J. Bloch J. I. Hastings A. K. Bourque J. R. Cadena E. A. Herrera F. A. Polly P. D. Jaramillo C. A. 2009 Giant boid snake from the Palaeocene neotropics reveals hotter past equatorial temperatures. Nature 457, 717–714 10.1038/nature07671 (doi:10.1038/nature07671) 19194448

[34] Legendre S. 1986 Analysis of mammalian communities from the late Eocene and Oligocene of southern France. Palaeovertebrata 16, 191–212

[35] Valverde J. A. 1967 Estructura de una comunidad de vertebrados terrestres. Monografías de la Estación Biológica de Doñana 1, 1–129

[36] Foley J. Levis S. Costa M. Cramer W. Pollard D. 2008 Incorporating dynamic vegetation cover within global climate models. Ecol. Appl. 10, 1620–1632 10.1890/1051-0761(2000)010[1620:IDVCWG]2.0.CO;2 (doi:10.1890/1051-0761(2000)010[1620:IDVCWG]2.0.CO;2)

[37] Schneider B. Schneider R. 2009 Palaeoclimate: global warmth with little extra CO_2_. Nat. Geosci. 3, 6–7 10.1038/ngeo736 (doi:10.1038/ngeo736)

[38] Royer D. 2001 Stomatal density and stomatal index as indicators of paleoatmospheric CO_2_ concentration. Rev. Palaeobot. Palynol. 114, 1–28 10.1016/S0034-6667(00)00074-9 (doi:10.1016/S0034-6667(00)00074-9) 11295163

[39] Maurer B. A. 1999 Untangling ecological complexity. Chicago, IL: University of Chicago Press

[40] Salick J. Cellinese N. Knapp S. 1997 Indigenous diversity of cassava: generation, maintenance, use and loss among the Amuesha, Peruvian upper Amazon. Econ. Bot. 51, 6–19 10.1007/BF02910400 (doi:10.1007/BF02910400)

[41] Salick J. Ross N. 2009 Traditional peoples and climate change introduction. Global Environ. Change Hum. Policy Dimens. 19, 137–139

[42] Pyšek P. 2010 Disentangling the role of environmental and human pressures on biological invasions across Europe. Proc Natl Acad. Sci USA 107, 12 157–12 162 10.1073/pnas.1002314107 (doi:10.1073/pnas.1002314107) PMC2901442 20534543

[43] Warren M. S. 2001 Rapid responses of British butterflies to opposing forces of climate and habitat change. Nature 414, 65–69 10.1038/35102054 (doi:10.1038/35102054) 11689943

[44] Van Der Made J. 2005 La fauna del Pleistocene Europeo. In Homínidos: Las primeras ocupaciones de los continentes (ed. Carbonell E.), pp. 394–416 Barcelona, Spain: Ariel

[45] Manly B. F. J. 2007 Randomization, bootstrap, and Monte Carlo methods in biology. Boca Raton, FL: Chapman & Hall/ CRC

[46] Raup D. 1975 Taxonomic diversity estimation using rarefaction. Paleobiology 1, 333–342

[47] Carrasco M. Barnosky A. Graham R. Stepanova A. 2009 Quantifying the extent of North American mammal extinction relative to the pre-anthropogenic baseline. PLoS ONE 4, e8331 10.1371/journal.pone.0008331 (doi:10.1371/journal.pone.0008331) 20016820 PMC2789409

[48] Rahbek C. Gotelli N. J. Colwell R. K. Entsminger G. L. Rangel T. Graves G. R. 2007 Predicting continental-scale patterns of bird species richness with spatially explicit models. Proc. R. Soc. B 274, 165–174 10.1098/rspb.2006.3700 (doi:10.1098/rspb.2006.3700) PMC1685854 17148246

[49] Polly P. D. 2004 On the simulation of morphological shape: multivariate shape under selection and drift. Palaeo. Electr. 7.2.7A, 28p

[50] Frank D. Esper J. Zorita E. Wilson R. 2010 A noodle, hockey stick, and spaghetti plate: a perspective on high-resolution paleoclimatology. Wiley Interdiscip. Rev. Clim. Change 1, 507–516 10.1002/wcc.53 (doi:10.1002/wcc.53)

[51] Fritts H. 1976 Tree rings and climate. Caldwell, NJ: Blackburn Press

[52] D'Arrigo R. Wilson R. Liepert B. Cherubini P. 2008 On the ‘divergence problem’ in northern forests: a review of tree-ring evidence and possible causes. Global Planet. Change 60, 289–305 10.1016/j.gloplacha.2007.03.004 (doi:10.1016/j.gloplacha.2007.03.004)

[53] Loehle C. 2009 A mathematical analysis of the divergence problem in dendroclimatology. Clim. Change 94, 233–245 10.1007/s10584-008-9488-8 (doi:10.1007/s10584-008-9488-8)

[54] Esper J. Frank D. 2009 Divergence pitfalls in tree-ring research. Clim. Change 94, 261–266 10.1007/s10584-009-9594-2 (doi:10.1007/s10584-009-9594-2)

[55] Esper J. Büntgen U. Frank D. Nievergelt D. Liebhold A. 2007 1200 years of regular outbreaks in alpine insects. Proc. R. Soc. B 274, 671–679 10.1098/rspb.2006.0191 (doi:10.1098/rspb.2006.0191) PMC2197206 17254991

[56] Cook E. R. Pederson N. 2010 Uncertainty, emergence, and statistics in dendrochronology. In Dendroclimatology: progress and prospects (eds Hughes M. K. Diaz H. Swetnam T. W.), pp. 77–112 Berlin, Germany: Springer Verlag

[57] Cook E. R. Cole J. 1991 Predicting the response of forests in eastern North America to future climatic change. Clim. Change 19, 271–282 10.1007/BF00140166 (doi:10.1007/BF00140166).

[58] Bruzgul J. E. Long W. Hadly E. A. 2005 Temporal response of the tiger salamander (*Ambystoma tigrinum*) to 3000 years of climatic variation. BMC Ecol. 5, 7 10.1186/1472-6785-5-7 (doi:10.1186/1472-6785-5-7) 16159383 PMC1249562

[59] Wolfe J. 1995 Paleoclimatic estimates from Tertiary leaf assemblages. Ann. Rev. Earth Planet. Sci. 23, 119–142 10.1146/annurev.ea.23.050195.001003 (doi:10.1146/annurev.ea.23.050195.001003)

[60] Imbrie J. Kipp N. 1971 A new micropaleontological method for quantitative paleoclimatology: application to a Late Pleistocene Caribbean core. In The late Cenozoic glacial ages (ed. Turekian K. K.), pp. 71–181 New Haven, CT: Yale University Press

[61] Bryson R. Kutzbach J. 1974 On the analysis of pollen-climate canonical transfer functions. Quat. Res. 4, 162–174 10.1016/0033-5894(74)90005-2 (doi:10.1016/0033-5894(74)90005-2)

[62] Janis C. M. Fortelius M. 1988 On the means whereby mammals achieve increased functional durability of their dentitions, with special reference to limiting factors. Biol. Rev. 63, 197–230 10.1111/j.1469-185X.1988.tb00630.x (doi:10.1111/j.1469-185X.1988.tb00630.x) 3042033

[63] White T. 1959 The endocrine glands and evolution, no. 3: os cementum, hypsodonty, and diet. Contrib. Museum Paleo. Univ. Mich. 13, 211–265

[64] Pfretzschner H. 1992 Enamel microstructure and hypsodonty in large mammals. In Structure, function and evolution of teeth (eds Smith P. Tchernov E.), pp. 147–162 London, UK: Freund Publishing House

[65] Van Valen L. 1960 A functional index of hypsodonty. Evolution 14, 531–532 10.2307/2406003 (doi:10.2307/2406003)

[66] Tummers M. Thesleff I. 2003 Root or crown: a developmental choice orchestrated by the differential regulation of the epithelial stem cell niche in the tooth of two rodent species. Development 130, 1049–1057 10.1242/dev.00332 (doi:10.1242/dev.00332) 12571097

[67] Solounias N. Fortelius M. Freeman P. 1994 Molar wear rates in ruminants: a new approach. Ann. Zool. Fenn. 31, 219–227

[68] Eronen J. T. Puolamäki K. Liu L. Lintulaakso K. Damuth J. Janis C. Fortelius M. 2010 Precipitation and large herbivorous mammals. I. Estimates from present-day communities. Evol. Ecol. Res. 12, 217–233

[69] Eronen J. T. Puolamäki K. Liu L. Lintulaakso K. Damuth J. Janis C. Fortelius M. 2010 Precipitation and large herbivorous mammals. II. Applications to fossil data. Evol. Ecol. Res. 12, 235–248

[70] Damuth J. 2001 Reconstructing mean annual precipitation, based on mammalian dental morphology and local species richness. In EEDEN program plenary workshop on Late Miocene to Early Pliocene environments and ecosystems (eds Agustí J. Oms O.), pp. 23–24 Brussels, Belgium: European Science Foundation

[71] Polly P. D. 2010 Tiptoeing through the trophics: geographic variation in carnivoran locomotor ecomorphology in relation to environment. In Carnivoran evolution: new views on phylogeny, form, and function (eds Goswami A. Friscia A.), pp. 347–410 Cambridge, UK: Cambridge University Press

[72] Elith J. 2006 Novel methods improve prediction of species' distributions from occurrence data. Ecography 29, 129–151 10.1111/j.2006.0906-7590.04596.x (doi:10.1111/j.2006.0906-7590.04596.x)

[73] Peterson A. 2001 Predicting species' geographic distributions based on ecological niche modeling. Condor 103, 599–605 10.1650/0010-5422(2001)103[0599:PSGDBO]2.0.CO;2 (doi:10.1650/0010-5422(2001)103[0599:PSGDBO]2.0.CO;2)

[74] Waltari E. Hijmans R. Peterson A. Nyári Á. Perkins S. Guralnick R. 2007 Locating Pleistocene refugia: comparing phylogeographic and ecological niche model predictions. PLoS ONE 2, e563 10.1371/journal.pone.0000563 (doi:10.1371/journal.pone.0000563) 17622339 PMC1905943

[75] Peterson A. T. Ortega-Huerta M. A. Bartley J. Sánchez-Cordero V. Soberón J. Buddemeier R. H. Stockwell D. R. B. 2002 Future projections for Mexican faunas under global climate change scenarios. Nature 416, 626–629 10.1038/416626a (doi:10.1038/416626a) 11948349

[76] Nogués Bravo D. 2009 Predicting the past distribution of species climatic niches. Global Ecol. Biogeogr. 18, 521–531 10.1111/j.1466-8238.2009.00476.x (doi:10.1111/j.1466-8238.2009.00476.x)

[77] Heikkinen R. K. Luoto M. Araujo M. B. Virkkala R. Thuiller W. Sykes M. T. 2006 Methods and uncertainties in bioclimatic envelope modelling under climate change. Prog. Phys. Geogr. 30, 751–777 10.1177/0309133306071957 (doi:10.1177/0309133306071957)

[78] Araújo M. Guisan A. 2006 Five (or so) challenges for species distribution modelling. J. Biogeogr. 33, 1677–1688 10.1111/j.1365-2699.2006.01584.x (doi:10.1111/j.1365-2699.2006.01584.x)

[79] Pearman P. B. Guisan A. Broennimann O. Randin C. F. 2007 Niche dynamics in space and time. Trends Ecol. Evol. 23, 149–158 10.1016/j.tree.2007.11.005 (doi:10.1016/j.tree.2007.11.005) 18289716

[80] Varela S. Rodríguez J. Lobo J. M. 2009 Is current climatic equilibrium a guarantee for the transferability of distribution model predictions? A case study of the spotted hyena. J. Biogeogr. 36, 1645–1655 10.1111/j.1365-2699.2009.02125.x (doi:10.1111/j.1365-2699.2009.02125.x)

[81] Polly P. D. Eronen J. T. 2011 Mammal associations in the Pleistocene of Britain: implications of ecological niche modelling and a method for reconstructing palaeoclimate. In The ancient human occupation of Britain (eds Ashton N. Lewis S. Stringer C.), pp. 279–304 London, UK: Elsevier

[82] Vieites D. Nieto-Román S. Wake D. 2009 Reconstruction of the climate envelopes of salamanders and their evolution through time. Proc Natl Acad. Sci USA 106, 19 715–19 722 10.1073/pnas.0902956106 (doi:10.1073/pnas.0902956106) PMC2780936 19887643

[83] Carstens B. Richards C. 2007 Integrating coalescent and ecological niche modeling in comparative phylogeography. Evolution 61, 1439–1454 10.1111/j.1558-5646.2007.00117.x (doi:10.1111/j.1558-5646.2007.00117.x) 17542851

[84] Maguire K. Stigall A. 2009 Using ecological niche modeling for quantitative biogeographic analysis: a case study of Miocene and Pliocene equinae in the great plains. Paleobiology 35, 587–611 10.1666/0094-8373-35.4.587 (doi:10.1666/0094-8373-35.4.587)

[85] Hendricks J. Lieberman B. Stigall A. 2008 Using GIS to study palaeobiogeographic and macroevolutionary patterns in soft-bodied Cambrian arthropods. Palaeogeogr. Palaeoclim. Palaeoecol. 264, 163–175 10.1016/j.palaeo.2008.04.014 (doi:10.1016/j.palaeo.2008.04.014)

[86] Gilman S. Urban M. Tewksbury J. Gilchrist G. Holt R. 2010 A framework for community interactions under climate change. Trends Ecol. Evol. 25, 325–331 10.1016/j.tree.2010.03.002 (doi:10.1016/j.tree.2010.03.002) 20392517

[87] Stenseth N. 2004 The effect of climatic forcing on population synchrony and genetic structuring of the Canadian lynx. Proc. Natl Acad. Sci. USA 101, 6056–6061 10.1073/pnas.0307123101 (doi:10.1073/pnas.0307123101) 15067131 PMC395922

[88] Klein D. Meldgaard M. Fancy S. 1987 Factors determining leg length in *Rangifer tarandus*. J. Mammal. 68, 642–655 10.2307/1381597 (doi:10.2307/1381597)

[89] Muray D. Larivière S. 2002 The relationship between foot size of wild canids and regional snow conditions: evidence for selection against a high footload? J. Zool. 256, 289–299 10.1017/S095283690200033X (doi:10.1017/S095283690200033X)

[90] Labandeira C. Dilcher D. Davis D. Wagner D. 1994 Ninety-seven million years of angiosperm–insect association: paleobiological insights into the meaning of coevolution. Proc Natl Acad. Sci USA 91, 12 278–12 282 10.1073/pnas.91.25.12278 (doi:10.1073/pnas.91.25.12278) PMC45420 11607501

[91] Labandeira C. 1997 Insect mouthparts: ascertaining the paleobiology of insect feeding strategies. Ann. Rev. Ecol. Syst. 28, 153–193 10.1146/annurev.ecolsys.28.1.153 (doi:10.1146/annurev.ecolsys.28.1.153)

[92] Wilf P. Labandeira C. 1999 Response of plant-insect associations to Paleocene-Eocene warming. Science 284, 2153–2156 10.1126/science.284.5423.2153 (doi:10.1126/science.284.5423.2153) 10381875

[93] Labandeira C. Johnson K. Wilf P. 2002 Impact of the terminal Cretaceous event on plant–insect associations. Proc Natl Acad. Sci USA 99, 2061–2066 10.1073/pnas.042492999 (doi:10.1073/pnas.042492999) 11854501 PMC122319

[94] Carpenter S. R. 2002 Ecological futures: building an ecology of the long now. Ecology 83, 2069–2083

[95] Kerr J. T. Kharouba H. M. Currie D. J. 2007 The macroecological contribution to global change solutions. Science 316, 1581–1584 10.1126/science.1133267 (doi:10.1126/science.1133267) 17569854

[96] Wake M. H. 2008 Integrative biology: science for the 21st century. Bioscience 58, 349–353 10.1641/B580410 (doi:10.1641/B580410)

[97] Wiemann M. Manchester S. Dilcher D. Hinojosa L. Wheeler E. 1998 Estimation of temperature and precipitation from morphological characters of dicotyledonous leaves. Am. J. Bot. 85, 1796–1802 10.2307/2446514 (doi:10.2307/2446514) 21680340

[98] Kowalski E. Dilcher D. 2003 Warmer paleotemperatures for terrestrial ecosystems. Proc Natl Acad. Sci USA 100, 167–170 10.1073/pnas.232693599 (doi:10.1073/pnas.232693599) 12493844 PMC140915

[99] Wing S. L. 2005 Transient floral change and rapid global warming at the Paleocene–Eocene boundary. Science 310, 993–996 10.1126/science.1116913 (doi:10.1126/science.1116913) 16284173

[100] Greenwood D. 2005 Leaf form and the reconstruction of past climates. New Phytol. 166, 355–357 10.1111/j.1469-8137.2005.01380.x (doi:10.1111/j.1469-8137.2005.01380.x) 15819898

[101] Uhl D. Mosbrugger V. 1999 Leaf venation density as a climate and environmental proxy: a critical review and new data. Palaeogeogr. Palaeoclimat. Palaeoecol. 149, 15–26 10.1016/S0031-0182(98)00189-8 (doi:10.1016/S0031-0182(98)00189-8)

[102] Woodward F. 1987 Stomatal numbers are sensitive to increases in CO_2_ from pre-industrial levels. Nature 327, 617–618 10.1038/327617a0 (doi:10.1038/327617a0)

[103] McElwain J. Chaloner W. 1995 Stomatal density and index of fossil plants track atmospheric carbon dioxide in the Palaeozoic. Ann. Bot. 76, 389–395 10.1006/anbo.1995.1112 (doi:10.1006/anbo.1995.1112)

[104] Makarieva A. M. Gorshkov V. G. Li L. 2005 Gigantism, temperature and metabolic rate in terrestrial poikilotherms. Proc. R. Soc. B 272, 2325–2328 10.1098/rspb.2005.3223 (doi:10.1098/rspb.2005.3223) PMC1560189 16191647

[105] Makarieva A. Gorshkov V. Li B. 2005 Temperature-associated upper limits to body size in terrestrial poikilotherms. Oikos 111, 425–436 10.1111/j.1600-0706.2005.14095.x (doi:10.1111/j.1600-0706.2005.14095.x)

[106] Gambaryan P. 1974 How mammals run: anatomical adaptations. New York, NY: Halsted Press

[107] Schmidt-Nielsen K. 1984 Scaling: why is animal size so important? Cambridge, UK: Cambridge University Press

[108] Smith F. Lyons S. Ernest S. Jones K. Kaufman D. Dayan T. Marquet P. Brown J. Haskell J. 2003 Body mass of late quaternary mammals. Ecology 84, 3403–3403 10.1890/02-9003 (doi:10.1890/02-9003)

[109] Rodríguez M. Olalla-Tárraga M. Hawkins B. 2008 Bergmann's rule and the geography of mammal body size in the western hemisphere. Global Ecol. Biogeogr. 17, 274–283 10.1111/j.1466-8238.2007.00363.x (doi:10.1111/j.1466-8238.2007.00363.x)

